# Healthy Lifestyle and Professional Identity in Nursing Students: A Scoping Review of Their Interrelationships

**DOI:** 10.3390/nursrep16040121

**Published:** 2026-04-02

**Authors:** Marelle Grünthal-Drell, Inge Timoštšuk, Martin Argus

**Affiliations:** 1 Chair of Nursing, Tallinn Health University of Applied Sciences, 13418 Tallinn, Estonia; martin.argus@ttk.ee; 2 School of Educational Sciences, Tallinn University, 10120 Tallinn, Estonia; inge.timostsuk@tlu.ee

**Keywords:** nursing students, professional identity, healthy lifestyle, self-care, professional self-concept, well-being

## Abstract

**Background:** Professional identity (PI) formation is a central developmental process associated with students’ well-being and ability to cope with professional demands. Healthy lifestyle (HL) and self-care are recognised as resources for sustaining long-term professional engagement. Although both PI formation and HL are considered important in nursing education, their interrelationship remains insufficiently understood. **Objective:** This review aimed to map and synthesise the existing literature on nursing students’ PI formation and its relationship with HL. **Methods:** A scoping review was conducted following the Arksey and O’Malley framework, Joanna Briggs Institute guidance, and PRISMA-ScR reporting standards. A systematic search was performed in Web of Science, Scopus, MEDLINE, and PubMed for peer-reviewed studies published 2015–2025. **Results:** Twelve sources met the inclusion criteria. The relationship between PI and HL is complex, indirect, and not yet clearly conceptualised. Rather than being defined through direct behavioural pathways, it appears to be mediated through mental well-being and related psychosocial aspects, as well as contextual influences. Tensions were identified between expectations of nurses as health role models and students’ lived behaviours. A well-developed PI may function as a protective resource against maladaptive coping and support-adaptive responses to academic and clinical stress. **Conclusions:** Both PI and HL are predominantly conceptualised as dynamic and contextually embedded processes. More integrative approaches addressing both behavioural and psychosocial dimensions are needed. Future research should adopt conceptually coherent and methodologically balanced designs across diverse educational contexts.

## 1. Introduction

Increasing workloads, emotional demands, and changing societal expectations place increasing pressure on nurses, highlighting the importance of their well-being [1,2]. A strong professional identity (PI) has been associated with greater commitment to the profession [3,4], while a healthy and engaged nursing workforce contributes to improved patient outcomes, reduced burnout and absenteeism, and enhanced organisational performance [2,5]. Within nursing, well-being is understood as a multidimensional concept. The American Association of Colleges of Nursing (AACN) defines wellness as a state characterised by emotional stability, including effective coping and the ability to form satisfying relationships, together with physical health, such as engagement in physical activity, healthy eating, and adequate sleep [6]. In parallel, the World Health Organization (WHO) defines a healthy lifestyle (HL) as a way of living that supports long-term well-being [7], encompassing behaviours and habits that promote physical, mental, and social well-being and prevent serious illness [8,9]. In this review, HL is understood broadly to include both health-related behaviours and related well-being dimensions.

Professional identity in nursing refers to a nurse’s self-concept and self-perception as a competent, ethical, and trustworthy practitioner, developed through the internalisation of professional values, norms, and expectations during nursing education [10,11]. Both, PI and HL, are shaped during nursing education and may influence how students adopt and sustain health-promoting behaviours.

National and international nursing standards emphasise the importance of nurses as professional role models, with their own lifestyle playing a key role in reinforcing public health messages and professional credibility [12,13,14]. As the largest group of healthcare professionals, nurses are expected to model health-promoting behaviours [1,3]. Nurses’ own health behaviours have been shown to influence their role in health promotion and their credibility as role models for patients [3,15]. Accordingly, maintaining HL is considered important not only for nurses’ personal well-being but also for the effectiveness and trustworthiness of healthcare organisations [12]. These principles align with patient-centred and health-promoting approaches, which reflect core nursing values, including the modelling of nurses’ own health behaviours [2,15]. Health behaviours refer to actions undertaken to promote, protect, or maintain health [16], while lifestyle can be understood as a system of daily habits and behavioural patterns influencing health status [9,17]. HL typically includes regular physical activity, a balanced diet, adequate sleep, and avoidance of harmful behaviours such as smoking and excessive alcohol use [8,17]. In addition, stress management and personal responsibility for health are important components, particularly in nursing, where self-care among nurses and nursing students is essential for maintaining both personal well-being and professional functioning [6,17,18,19]. Inadequate self-care may lead to reduced performance, increased errors, and compromised patient safety, highlighting its relevance as both a personal and professional responsibility [18].

Despite the recognised importance of HL, evidence suggests that many nurses struggle to maintain their own health and well-being. Physical activity levels remain low [20], and unhealthy behaviours may reduce nurses’ effectiveness as health promoters [21]. Nurses have also been reported to lack sufficient knowledge of lifestyle-related factors and recovery strategies, increasing vulnerability to burnout, stress, and related health problems [22]. Similar challenges have also been observed among nursing students, including overweight and obesity, low levels of physical activity, inadequate sleep, and other unhealthy lifestyle behaviours. Sedentary habits and poor dietary choices are of particular concern, as they may negatively affect both students’ personal health and their ability to provide effective health guidance to future patients [23,24,25]. Students’ beliefs, attitudes, and behaviours may also influence the clinical care they provide [23], highlighting the importance of developing HL practices during training. Accordingly, nursing students should be supported in improving their own lifestyles before advising patients on health-related behaviours. This underscores the importance of integrating HL development into nursing education, where students are expected not only to promote health in others but also to embody health-promoting behaviours themselves [3,25,26].

A growing body of research has examined health behaviours and well-being among nursing students and nurses [14,20,21,22,23,24,25,26]. However, these constructs are often addressed separately, and their relationship with PI formation is rarely examined explicitly. Furthermore, HL is sometimes represented indirectly through broader well-being-related aspects rather than clearly defined lifestyle behaviours. Consequently, the conceptual boundaries of HL remain variable and sometimes unclear, making it difficult to interpret how HL relates to PI formation in nursing education. Despite increasing attention to these issues, existing studies and reviews have not yet provided a comprehensive synthesis of how HL is conceptualised in relation to PI formation among nursing students. Understanding how nursing education can support the development of professional identity while strengthening a healthy lifestyle is therefore of increasing importance. Linking HL with PI may help prepare students for the demands of a stressful profession, support their self-care abilities, and contribute to high-quality and safe patient care.

In response to this gap, this scoping review aimed to map and synthesise the existing literature on nursing students’ professional identity formation and its relationships with healthy lifestyles. This review addressed the following research question: how are the relationship between professional identity formation and healthy lifestyle conceptualised in the literature on nursing students? The review further addressed the following sub-questions: (1) Which key concepts and study contexts are reported in the literature when examining this relationship? (2) How are professional identity and healthy lifestyle described and conceptualised in nursing education? (3) Which aspects of a healthy lifestyle are associated with professional identity development among nursing students?

## 2. Materials and Methods

### 2.1. Study Design

This scoping review followed the methodological framework proposed by Arksey and O’Malley [27] and the Joanna Briggs Institute (JBI) guidance [28], and was reported according to the PRISMA-ScR guidelines [29]. No protocol was registered for this review; however, established methodological and reporting frameworks were followed to ensure transparency and rigour.

### 2.2. Eligibility Criteria

Eligibility criteria were developed using the Population–Concept–Context (PCC) framework.

Inclusion criteria:

– Types of studies: empirical research (qualitative, quantitative, or mixed methods) and review articles (systematic, scoping, or narrative reviews).
– Participants: undergraduate nursing students.
– Concept: studies addressing nursing students’ PI formation and HL, including their relationships between these concepts.
– Context: nursing education in formal academic environments, such as universities and professional nursing schools.
– Publication criteria: peer-reviewed full-text articles published in English between 2015 and 2025. Only open-access articles were included.
– Studies addressing HL either directly (e.g., health-related behaviours) or indirectly in relation to mental well-being and related psychosocial aspects.

Review articles were included to map existing syntheses, identify conceptual perspectives, and highlight research gaps, as well as to provide contextual and conceptual insights into the relationship between PI and HL. Although they were not included in the primary synthesis of empirical findings, they informed the identification of key concepts and supported the overall conceptual understanding of the topic. The time frame (2015–2025) was selected to capture contemporary evidence reflecting recent developments in nursing education, including evolving perspectives on PI formation and increasing attention to health-related behaviours and student well-being.

Exclusion criteria: editorials, commentaries, conference abstracts, theses, and grey literature (e.g., reports); studies that did not examine any relationship between nursing students’ PI formation and HL, either directly or indirectly in relation to mental well-being and related psychosocial aspects (e.g., studies addressing general health behaviours or well-being-related aspects without reference to PI).

### 2.3. Information Sources

The search was conducted in December 2025 using the following databases: Web of Science (Clarivate), Scopus (Elsevier), MEDLINE (via EBSCO), and PubMed. These databases were selected for their broad coverage of health sciences and educational research. Web of Science and Scopus index a wide range of interdisciplinary journals, while PubMed and MEDLINE focus on the biomedical and health-related literature, thereby enhancing methodological rigour. Although PubMed includes MEDLINE-indexed records, MEDLINE (via EBSCO) and PubMed were searched as separate sources due to differences in search interfaces and access points, allowing for more complete retrieval of relevant and recent publications. This combination of databases was considered appropriate for capturing literature on nursing students’ PI and HL, including both health-related behaviours and well-being-related aspects, within formal nursing education contexts in European and Nordic settings.

### 2.4. Search Strategy

Search terms based on key concepts related to PI and HL were combined using the Boolean operators AND and OR. A comprehensive search strategy was developed using a wide range of keywords and synonyms related to PI, HL, and nursing students to maximise sensitivity and ensure adequate coverage of the relevant literature. The search strategy was adapted to the specific interface and indexing structure of each database. Both British (e.g., behaviour) and American (e.g., behaviour) English spelling variants were included in the search strategy to maximise sensitivity and ensure comprehensive retrieval of relevant studies.

The full electronic search strategy for the Scopus database is provided in Appendix A.1. For MEDLINE and PubMed, searches incorporated controlled vocabulary (MeSH terms) alongside free-text keywords to ensure systematic, concept-based retrieval. The use of MeSH terms enhanced precision and supported the identification of related, broader, and narrower concepts. The complete electronic search strategy for MEDLINE (via EBSCO) is provided in Appendix A.2.

### 2.5. Selection of Sources of Evidence

All identified records were imported into EndNote (Version 20, Clarivate Analytics, Philadelphia, PA, USA), and duplicates were removed. Titles and abstracts were screened independently by two reviewers (M.G.-D. and M.A.) according to the predefined eligibility criteria. Full-text articles deemed potentially relevant were retrieved and assessed against the inclusion and exclusion criteria. No disagreements occurred during the screening process, and the selection of studies was agreed by the reviewers. The selection process followed the PRISMA-ScR checklist [30], which was applied throughout the planning and reporting stages to ensure that key methodological components were clearly described. This supported structured and transparent reporting and alignment with JBI guidance. A completed checklist is provided in Supplementary Materials (Table S1: PRISMA_2020_checklist). Review articles were screened using the same eligibility criteria as primary empirical studies to ensure consistency in study selection. During full-text assessment, articles were excluded if they did not examine any relationship between nursing students’ PI formation and HL, either directly or indirectly in relation to mental well-being and related psychosocial aspects. The main reasons for exclusion at this stage included studies addressing general health behaviours or well-being without reference to PI. Both empirical studies and review articles were retained to map existing evidence and theoretical perspectives; however, only empirical studies were included in the primary synthesis of results.

### 2.6. Data Charting and Synthesis

Data extraction was performed by the first author using a standardised data charting form developed specifically for this review (Appendix A.3). Extracted information was organised into summary tables addressing the review questions. Microsoft Word was used to organise the extracted data into structured tables. The extracted data included author(s), year of publication, study design, participant characteristics, study contexts (including country and educational and clinical settings), keywords (reflecting key concepts), study aims, and outcomes relevant to review question 1. The conceptualisation of PI and HL was charted to address review question 2, while their relationships were examined in relation to the main research question. HL aspects were charted to address review question 3.

Data were analysed descriptively and synthesised narratively using a structured thematic approach. The synthesis followed an iterative process involving familiarisation with the extracted data, grouping of studies based on similarities in concepts, definitions, and reported outcomes, and the identification of recurring patterns and themes. Concepts related to PI, HL, and their interrelationships were coded and organised into thematic categories. Conceptual mapping was used to compare findings across studies and to identify similarities and differences in how these concepts were described and related within nursing education contexts. The extraction process was guided by a standardised charting form. Other authors reviewed and verified the extracted data and thematic groupings to enhance consistency and credibility.

Findings from review articles were charted separately and organised into a separate table to identify key concepts and methodological approaches relevant to the current study. For each included review article, data were charted on authors, year of publication, review type, focus, scope, and reported outcomes. Their inclusion supported the contextualisation and interpretation of the findings in the Discussion and contributed to a more comprehensive understanding of the topic.

In accordance with JBI guidance [28] for scoping reviews, no formal critical appraisal of individual sources of evidence was conducted, as the purpose of this review was to map the existing literature. Instead, the review focused on providing an overview of key concepts and approaches and identifying existing knowledge gaps.

## 3. Results

**Figure 1:**
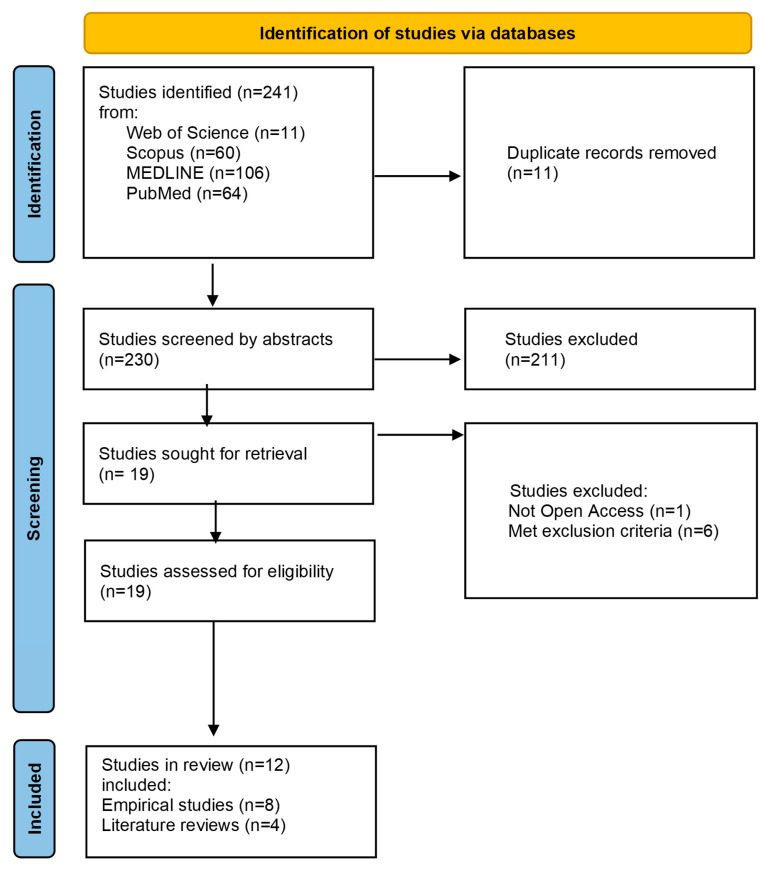
PRISMA flow diagram of the literature search, adapted from [30].

A total of 241 records were identified through database searches, including 106 from MEDLINE, 64 from PubMed, 60 from Scopus, and 11 from Web of Science. After screening and eligibility assessment, 12 studies published between 1 March 2017 and 7 November 2025 were included in the final analysis. Of these, eight were empirical studies (five quantitative and three qualitative) and four were review articles. The study selection process is presented in the PRISMA flow diagram (Figure 1).

### 3.1. Key Concepts and Study Contexts in the Existing Literature

All quantitative studies were conducted in Asia, including four studies from China and one from Indonesia, and involved a total of 31 higher education institutions offering nursing education. In contrast, all qualitative studies were conducted in Europe and included universities with nursing faculties in Denmark and England, as well as two vocational nursing schools in Finland. Quantitative studies reported sample sizes ranging from 286 to 1332 participants, whereas qualitative studies involved smaller samples, ranging from 26 to 29 participants. Table 1 provides a concise overview of the characteristics of the included studies, including year of publication, authors, study design, participants, context, country, keywords, study aims, and key findings.

Several studies were conducted in demanding educational and societal contexts characterised by elevated academic pressure, psychological strain, and broader social challenges affecting nursing education and the nursing profession. One study situated in the immediate post-pandemic period highlighted nursing students’ experiences of high academic and emotional demands within a context of wider social challenges influencing nursing education [34]. Another study reported increased stress and existential concerns within the healthcare sector alongside changing societal attitudes toward nursing as a profession [35]. A third study was conducted in higher education settings characterised by high academic and clinical workloads, as well as a high prevalence of smartphone addiction among nursing students from two medical universities [37]. Two studies focused specifically on undergraduate nursing students in their final year and were situated in transitional educational and clinical contexts associated with the shift from student to professional roles. One study examined students’ experiences of this transition, with particular emphasis on adaptation to internal and external stimuli [38]. The other study, conducted within a university college and during clinical placement in a university hospital, highlighted the role of students’ everyday experiences in shaping self-understanding and PI development [36]. Two further studies were situated in professional and educational contexts influencing nursing students’ PI development and health-related behaviours. One study examined moral tensions between students’ smoking behaviours and professional expectations in smoke-free healthcare settings [31]. The other study was conducted within a broader societal and political context characterised by strong expectations for nurses to act as role models for health-related behaviours despite challenging working conditions [33]. The final study was conducted in a nursing education context lacking a unified self-care framework, where nursing as a profession was characterised by a high risk of stress and burnout [32].

Of the 12 included sources, four were review articles (two systematic reviews, one scoping review, and one narrative review). These were identified alongside empirical studies to provide background and conceptual framing but were not included in the primary synthesis of empirical findings. An overview of the included review articles, including authors, year of publication, review type, focus, scope, and reported outcomes, is presented in Table 2.

### 3.2. Conceptualisations of Professional Identity, Healthy Lifestyle, and Their Interrelationships in Nursing Education

Table 3 provides a concise overview of the conceptualisations of nursing students’ PI and HL, as well as their interrelationships in the included literature. These conceptualisations are further elaborated below in the following sections.

#### 3.2.1. Conceptualisations of Nursing Students’ Professional Identity

Across the included literature, nursing students’ PI was conceptualised in multiple, partly overlapping ways, commonly as a dynamic, developmental, and relational process that evolves throughout nursing education [31,34,36]. PI was described as the adoption and internalisation of a socially defined professional role, involving identification with the nursing profession and commitment to its values. In some cases, descriptions also included elements of professional self-concept, such as evaluation of the profession [35,37].

Several studies conceptualised PI as a multidimensional construct encompassing cognitive, emotional, and social dimensions. These included processes such as social comparison, reflection, autonomy in career choice, and sensitivity to social influence [34,35]. In addition, some studies described elements such as professional self-belief, perceived benefits of commitment to the profession, and risks associated with leaving it, which were described in relation to professional self-concept [34,35]. Identification with the nursing role was also described as including professional self-image, certainty regarding career choice, independence in professional decision-making, and engagement in social modelling and self-reflection. Elements such as professional self-image and certainty regarding career choice were described as components of professional self-concept within the broader framework of professional identity [31,35,38].

PI was portrayed as emerging and relatively unstable during nursing education and shaped through students’ everyday participation in educational and clinical practices [31,36]. PI was also described as students’ growing understanding of the goals, values, and societal significance of the nursing profession, alongside readiness to invest in professional development and future practice [37]. Studies reported that its development was influenced by students’ lived experiences, agency, and sense of belonging within communities of practice [35,36]. In some studies, aspects such as self-understanding were also highlighted [35,36], representing elements of professional self-concept.

Supportive educational and clinical environments were described as conditions for PI formation, particularly those characterised by ontological and psychological safety, constructive learning cultures, meaningful social interactions, and engagement with professional role models [35,36]. PI was also conceptualised through social and institutional expectations related to the nursing role, encompassing professional credibility, ethical responsibility, and alignment between professional values and behaviour [33]. Elements such as alignment between values and behaviour were described as aspects of professional self-concept within the broader framework of professional identity.

In several studies, PI was described as being associated with patient-centred, evidence-based, and empathetic care. Experienced nurses and educators were described as influential role models shaping students’ professional judgements and orientations through everyday interactions and observed behaviours [33,36].

PI was also conceptualised as the development of the self into a “healing instrument” and a role model for patients. In this context, self-development was described as involving the cultivation of deeper awareness and a so-called healing consciousness, associated with compassionate and effective care. Within this conceptualisation, elements such as self-development and self-care were described as supporting professional growth. 

Self-care, in particular, was described as a prerequisite for professional development rather than a direct component of professional identity, supporting holistic balance, including both physical and mental well-being [32]. PI development was described as a cyclical and ongoing process that unfolds throughout nursing education and provides a foundation for professional growth, career commitment, and the transition from student to professional nurse [31,37]. Some studies described elements related to professional self-concept as distinct but related components within the broader framework of professional identity development [35,36,37].

#### 3.2.2. Conceptualisations of Healthy Lifestyle

Across the included literature, HL was conceptualised at two levels: as a behavioural construct and as a broader construct examined in relation to mental well-being and related psychosocial aspects.

HL was described as a behavioural and observable construct, expressed through specific health behaviours and indicators. Visible manifestations of HL included body weight and smoking behaviour [33], while other health-related behaviours were not explicitly described in the included studies. Smoking was described as a behavioural expression of lifestyle and, in some contexts, as a normalised and controllable practice related to coping with stress and emotional demands. Some students also reported concerns that smoking cessation could challenge their sense of identity or make everyday coping more difficult [31]. These studies also reported that both nursing students and practising nurses were reported to adopt unhealthy behaviours as responses to stress and emotional demands [31,33]. Within this behavioural framing, role modelling of healthy behaviours was described as an individual choice rather than a professional obligation, with personal health behaviours considered part of the private sphere rather than an automatic indicator of professional competence or quality of care [31,33].

HL was also described as a holistic construct encompassing both physical and mental well-being. Within this perspective, HL included self-care as a multidimensional practice encompassing eight dimensions: physical, mental, emotional, and spiritual well-being, satisfying interpersonal relationships, life satisfaction, environmental awareness, and responsibility for one’s own health [32]. Findings indicated that health-related behaviours such as physical activity and nutrition were less frequently practised, while self-care activities were more often oriented towards emotional well-being among nursing students. Students were also reported to adopt unhealthy physical behaviours in response to academic demands and clinical stressors [32].

Several studies examined HL in relation to mental well-being and related psychosocial aspects rather than as a clearly defined behavioural concept. Meaning in life and self-care were described in relation to problematic smartphone use, which was associated with disrupted daily functioning, sleep disturbances, and reduced well-being among nursing students [37]. Meaning in life was also described in association with subjective well-being, hope, and optimism, and in relation to health-related behaviours and coping in stressful situations [35,37]. Lower levels of perceived meaning were associated with increased negative emotions and poorer mental health outcomes [35,37].

Other studies described meaning in life, sense of coherence, psychological well-being (psychological capital), and ontological security as factors associated with well-being, mental stability, coping, resilience, and learning capacity among nursing students [34,35,36,37,38]. In two studies, HL was examined in relation to these psychosocial constructs, including sense of coherence and ontological security, which were described in association with mental stability, stress management, and learning capacity in educational and clinical contexts [34,36]. Sense of coherence was also described as a health-related resource that could develop over time and was associated with more positive outlooks and mental health outcomes among nursing students [34]. These constructs were presented in relation to HL but were not described as direct expressions of lifestyle behaviour. In some studies, HL was also described as extending beyond health-related behaviours to include processes related to self-development, reflection, and balance across multiple domains of well-being [32].

#### 3.2.3. Interrelationships Between Professional Identity and Healthy Lifestyle Concepts

Across the included studies, interrelationships between PI and HL were described as multi-level and predominantly indirect, varying across educational and clinical contexts and according to students’ developmental stages. These relationships were described in relation to mental well-being and related psychosocial aspects, as well as broader contextual factors, including coping and self-development, rather than as clearly defined behavioural links. In some studies, the connection between PI and HL was also described in terms of professional values, role expectations, and role modelling within nursing education and practice.

In several studies, the relationship between PI and HL was described in relation to visible health behaviours. HL was framed as a moral and professional expectation, particularly in relation to behaviours such as body weight and smoking, which were described in relation to nurses’ professional credibility and authority as health promoters [33]. In this context, a visibly HL was described in relation to the perceived credibility of health advice, and PI was also associated with the ability to meet the physical demands of nursing work and maintain professional competence [33]. In contrast, nursing students did not consistently associate personal health behaviours with professional competence or quality of care and described these behaviours primarily as part of the private sphere rather than as an integral component of PI [31,33]. Personal health behaviours were presented as individual choices rather than professional obligations. Tension was described between PI and HL, particularly in relation to smoking, which was described as inconsistent with professional norms but also as a socially embedded behaviour associated with identity and belonging [31]. Although smoking was recognised as an unhealthy lifestyle behaviour, it was not described as directly conflicting with future professional practice or as a barrier to patient care or professional competence. Tension was also described between PI and smoker identity, the latter being associated with emotional significance, social embeddedness, and a sense of belonging and self-definition [31]. The role of mentors and the hidden curriculum was described as influencing both health-related behaviours and PI development. The behaviours and communication styles of experienced nurses were described as shaping students’ professional judgements and approaches to patient care [31,33,36]. In this context, alignment with observed practices, including smoking behaviours, formed part of students’ engagement within clinical environments. Smoking was described as facilitating social interaction and enabling contact with practising nurses who smoked during clinical placements [31].

In one study, the relationship between PI and HL was described in relation to holistic self-care. HL was conceptualised as self-care and self-development, encompassing practices related to balance across physical and mental well-being [32]. Within this perspective, self-care was described in association with self-awareness, reflective thinking, and resilience, as well as with students’ transition into professional roles in nursing. Insufficient self-care was described in relation to lower levels of emotional functioning and challenges in coping, which were also reported in relation to professional functioning and intentions to remain in the nursing profession. Self-care was further described as part of both personal and professional development among nursing students and practising nurses. Nursing care was described in relation to nurses’ own health and well-being, including aspects such as compassionate, safe, and responsible care, which were presented in relation to self-care and holistic approaches in nursing practice [32].

In several studies, relationships between PI and HL were described in relation to mental well-being and related psychosocial aspects. In particular, among nursing students, PI and HL were described in relation to coping-related behaviours. PI was described in relation to meaning in life and problematic smartphone use, with stronger perceptions of meaning and PI reported alongside lower levels of maladaptive behaviours such as excessive device use. Students who identified more strongly with their future professional role were reported to direct their efforts towards professional development rather than towards behaviours providing immediate gratification, such as excessive device use [37]. Meaning in life was described in relation to mental well-being and professional development, in association with life satisfaction and engagement with professional goals. Higher levels of perceived meaning were reported alongside more positive emotions and stronger PI. A clear sense of life purpose was described in relation to coping with professional challenges. In contrast, lower levels of perceived meaning were associated with increased negative emotions, poorer mental health, and difficulties in professional decision-making, including decision-related anxiety [35]. Sense of coherence (SoC) was described in relation to PI, with higher levels of SoC reported alongside more positive academic emotions, self-evaluation, and professional satisfaction. SoC was also described in association with nursing students’ mental stability and positive self-perception, as well as with the perception of nursing as a valuable and meaningful profession. Negative academic emotions were described as having indirect effects in relation to self-esteem [34]. Psychological capital was also described in relation to professional values, mental well-being, and intentions to enter the nursing workforce. It was further identified as a resource associated with effective coping and sustained engagement in professional development, with higher levels reported alongside stronger PI and increased intentions to work within the nursing profession [38]. HL was also described in relation to broader well-being-related contexts, including ontological and psychological safety and emotional well-being in educational and clinical contexts. Supportive environments were described in association with confidence, engagement in learning, and PI development, whereas the absence of such conditions was associated with emotional strain and reduced engagement in learning processes and PI formation [36].

In several studies, the relationship between health behaviours and PI was not described as a direct or expected link. PI was also described in association with coping in situations of stress and uncertainty, as well as with lower levels of perceived stress and negative emotions [37].

### 3.3. Healthy Lifestyle Aspects Associated with Professional Identity Development

Across the included studies, several aspects of a healthy lifestyle were described in association with professional identity development among nursing students. These aspects were grouped into three main areas: mental well-being-related aspects, physical and mental well-being-related aspects, and physical health behaviour-related aspects. While behavioural lifestyle factors were addressed in some studies, other studies focused on aspects related to mental well-being, including those examined in relation to psychosocial factors. Table 4 provides an overview of HL aspects associated with PI in the included studies.

Two studies identified physical health behaviour-related aspects of HL, focusing on visible health-related behaviours such as body weight and smoking [31,33]. These behaviours were associated with professional credibility and role expectations related to health role modelling. Smoking was also linked to coping-related practices embedded in social and identity contexts [31]. One study examined HL in relation to physical and mental well-being-related aspects, conceptualising HL as holistic self-care encompassing both physical and mental well-being and balance during nursing education [32]. Five studies identified mental well-being-related aspects of HL, expressed through meaning in life, sense of coherence, psychological capital, and ontological security [34,35,36,37,38]. These aspects were associated with emotional stability, coping, and professional development.

Existing literature reviews primarily addressed professional self-concept in nursing education. Three reviews synthesised themes related to its conceptualisation, influencing factors, measurement approaches, outcomes, and intervention strategies. These reviews described professional self-concept in relation to nursing student burnout, psychological health, well-being, engagement, and professional functioning, as well as educational and psychosocial strategies aimed at mitigating burnout. In contrast, one review examined nursing students’ experiences of stress, describing stress in relation to both adverse outcomes and learning and professional development.

## 4. Discussion

This scoping review aimed to map and synthesise the existing literature on nursing students’ professional identity formation and its relationship with a healthy lifestyle. The findings indicate that only a limited number of studies have addressed this relationship, reflecting a lack of integrated research on PI formation and HL in nursing education.

The literature suggests that HL is not consistently conceptualised as a distinct behavioural construct in relation to PI, but is more often examined through associated aspects such as self-care, coping, and well-being. The findings further indicate that the relationship between health behaviours and PI is not consistently described as a direct or expected link. Rather than representing a clearly defined behavioural pathway, the relationship between PI and HL appears to be indirect and context-dependent, and is described through mental well-being and related psychosocial aspects, as well as contextual factors. Current evidence indicates that this relationship is shaped by educational, clinical, and social contexts, as well as by students’ developmental stages. Both constructs, PI and HL, are predominantly conceptualised as dynamic, relational, and contextually embedded processes that evolve throughout nursing education. This shared conceptualisation may partly explain why their interrelationship is more frequently described through associated processes rather than through direct behavioural connections.

The analysis also revealed a clear geographical and methodological distribution in the literature: quantitative studies were predominantly conducted in Asian contexts, whereas qualitative studies were more frequently situated in European settings. Although challenges related to nursing education and practice are global, this distribution suggests that in-depth exploration of students’ subjective experiences remains regionally concentrated. Across study contexts, PI formation was most often examined in environments characterised by high academic and emotional demands, where stress, burnout, and uncertainty regarding future employment are common. This aligns with earlier reviews emphasising that professional development in nursing frequently occurs under demanding learning conditions, in which supportive structures and psychological resources are important [39,40]. This context may further highlight the importance of addressing HL and related supportive factors within nursing education, particularly those that can strengthen students’ capacity to cope with academic and clinical demands. In this regard, the present review contributes to a more integrated understanding of how such factors may be linked to PI development.

### 4.1. Conceptualisations of Professional Identity in Nursing Education

The findings of this review suggest that PI in nursing education can be understood as a complex and multidimensional process emerging through the interaction of individual, social, and contextual factors. This interpretation is consistent with previous reviews, which have described PI as a dynamic construct shaped by personal characteristics, emotional processes, and organisational conditions [40,41,42]. The included studies further indicate that PI formation is closely linked to processes of self-concept development, including self-evaluation, self-belief, and professional commitment. The observed overlap between PI and professional self-concept suggests that these constructs are not always clearly distinguished in the literature, but are often conceptualised as interconnected aspects of professional development. This may reflect the evolving nature of identity formation in nursing education, where cognitive, emotional, and social dimensions appear to be closely intertwined [34,35,38].

Evidence from the included studies indicates that PI develops through students’ active participation in educational and clinical environments. Experiences of belonging, agency, and interaction with professional role models appear to contribute to shaping students’ understanding of the nursing profession and their place within it [35,36]. This interpretation aligns with previous research emphasising the importance of learning environments and socialisation processes in PI formation [40,41]. In addition, the review highlights the relevance of supportive educational and clinical contexts, particularly those characterised by psychological and ontological safety, constructive learning cultures, and meaningful interpersonal interactions. Such conditions are described in the literature as being associated with students’ engagement in learning and the development of professional values and behaviours [35,36]. Although meaning in life and sense of life meaning are not identical constructs, they refer to closely related existential dimensions concerning the extent to which individuals perceive their lives as purposeful, coherent, and valuable. The included studies conceptualised and operationalised these constructs with some variation; however, they consistently converged in describing them as key psychological resources supporting mental well-being, coping, and professional development. For the purposes of this review, these related constructs are discussed under the broader conceptual umbrella of meaning in life to enhance clarity and coherence, while acknowledging the nuanced distinctions in their original theoretical and methodological applications. This synthesis may suggest that existential meaning functions as a central underlying process linking well-being and professional identity development in nursing students.

The role of experienced nurses and educators as role models further supports the view that PI is shaped through everyday practices and interactions rather than through formal instruction alone [33,36]. This may suggest that professional identity formation is not solely driven by curriculum content, but also by the implicit values and behaviours demonstrated within educational and clinical environments, highlighting the importance of supportive and reflective learning contexts.

The literature also suggests that PI is associated with professional values related to patient-centred, evidence-based, and empathetic care. At the same time, elements such as self-development and self-care are described in relation to professional growth, indicating that personal development processes may be closely linked to PI formation [32]. However, self-care is not consistently conceptualised as a direct component of PI, but rather as a related process supporting overall well-being and professional functioning. This distinction may be particularly relevant when considering the relationship between PI and HL. This interpretation is consistent with broader literature emphasising the importance of self-care and health-related responsibility in nursing, where maintaining personal well-being is closely linked to professional functioning [6,17,18,19].

Taken together, current evidence indicates that PI can be understood as an ongoing and context-dependent process rather than a fixed outcome. The cyclical and developmental nature of PI described in the included studies suggests that identity formation extends throughout nursing education and into professional practice [31,37].

### 4.2. Conceptualisation of a Healthy Lifestyle in Nursing Education

The findings of this review suggest that HL in nursing education can be conceptualised in two interrelated but distinct ways. On the one hand, HL is understood in line with its traditional definition as a set of health-related behaviours, including observable practices such as smoking and body weight [33]. On the other hand, the literature increasingly examines HL in relation to broader processes such as self-care, coping, and well-being. This duality suggests that, although behavioural aspects of HL are acknowledged, they are not consistently positioned as central components within the included literature on nursing education. Instead, visible behaviours tend to be interpreted through their social and professional meaning, particularly in relation to credibility and role expectations in nursing practice [31,33]. This may suggest that HL is framed less as a set of behaviours and more as a socially situated construct. At the same time, nursing students often describe these behaviours as part of the private sphere rather than as an inherent element of their educational or professional development, indicating a potential disconnect between personal lifestyle and professional expectations. This may also suggest that, within the included literature, healthy lifestyle is not consistently conceptualised as a health-promoting resource in its own right, but rather as an individual or contextual factor.

Current evidence indicates that HL is frequently associated with mental well-being and related psychosocial aspects, such as meaning in life, sense of coherence, and psychological capital, which are linked to well-being, coping, and adaptation within educational and clinical environments [34,35,36,37,38]. However, these constructs should not be interpreted as components of HL itself, but rather as related factors that may influence how health behaviours are understood and enacted. This distinction is important, as it reflects a broader conceptual shift in the literature from behaviour-oriented definitions of HL towards more experience-based and psychosocial interpretations. This shift may also contribute to the relative invisibility of concrete health behaviours within the literature.

In addition, self-care appears to occupy a central position within discussions of HL in the included literature on nursing education. While it is associated with balance and well-being, it is not consistently conceptualised as a behavioural component of HL, but rather as a related process supporting students’ functioning in demanding educational and clinical environments [32]. This may help explain why health-related behaviours such as physical activity and nutrition are less visible in the literature compared to well-being and psychosocial aspects. These patterns are consistent with previous reviews indicating that HL remains conceptually fragmented, with greater emphasis placed on aspects related to mental well-being and coping processes than on concrete health behaviours [39,41,42]. However, although the literature predominantly emphasises well-being-related dimensions, these are inherently supported by and intertwined with behavioural factors that remain underrepresented in current conceptualisations. This contrasts with broader frameworks, which highlight that emotional stability, effective coping, and the ability to form satisfying relationships are underpinned by health behaviours, including engagement in physical activity, healthy eating, and adequate sleep [6]. This discrepancy may indicate a gap between theoretical definitions of HL and its operationalisation within nursing education research.

Taken together, the literature suggests that, within nursing education, HL is not consistently conceptualised as a distinct behavioural construct but is often interpreted through well-being, psychosocial aspects, and contextual processes. This interpretation should be considered with caution, as it may obscure the role of concrete health behaviours in students’ well-being and functioning within educational and clinical settings.

### 4.3. Healthy Lifestyle Aspects Related to Professional Identity Development

Building on this conceptualisation, the literature further indicates that different aspects of HL are emphasised unevenly within nursing education, reflecting a divergence between behavioural, holistic, and psychosocial dimensions. Consistent with the earlier conceptualisation, behaviour-oriented approaches tend to focus on visible health-related indicators, such as body weight and smoking [31,33], further reflecting the emphasis on externally observable aspects of HL. However, such approaches appear to place limited emphasis on underlying psychological processes and are not consistently integrated into students’ lived experiences within nursing education. The literature further suggests that, although physical activity and nutrition are addressed within nursing curricula, they may be among the least consistently practised self-care behaviours [32]. At the same time, these findings are complemented by studies that adopt a more holistic perspective, conceptualising HL as a form of self-management that integrates both physical and mental well-being [32]. From this perspective, HL is understood as a dynamic process of maintaining balance in the context of academic and clinical demands, rather than as a set of discrete behaviours. Broader frameworks, such as that of the AACN [6], further extend this view by highlighting self-management across physical, emotional, social, spiritual, and professional domains. This may support a more integrative understanding of HL as a context-dependent and actively regulated process.

At the same time, the literature places a stronger and more consistent emphasis on mental well-being and related psychosocial aspects. Constructs such as meaning in life, sense of coherence, psychological capital, ontological security, emotional regulation, and resilience are frequently described as central factors associated with students’ well-being, coping, and engagement within educational and clinical contexts [34,35,36,37,38]. These aspects are often conceptualised as resources that support adaptive coping and emotional regulation, shaping how students may interpret and respond to stress. Psychological well-being and psychological capital have similarly been associated with effective functioning and potential indirect benefits for professional performance [36,38].

Taken together, this pattern suggests that, although HL encompasses behavioural, holistic, and psychosocial dimensions, the literature tends to privilege mental well-being and related psychosocial aspects over behavioural practices. This imbalance may contribute to the observed gap between knowledge, expectations, and everyday behaviours, and further reflects the broader conceptual fragmentation of HL within nursing education research.

### 4.4. Interrelationships Between Professional Identity and Healthy Lifestyle

The findings of this review suggest that the relationship between PI and HL in nursing education is complex and not yet conceptually well defined. Rather than being explicitly integrated, these constructs appear to be connected through overlapping processes related to personal development, well-being, and engagement within educational and clinical contexts. The literature indicates that, although PI and HL are often examined separately, they may intersect in ways that are not consistently articulated. This may reflect a broader lack of conceptual clarity regarding how health-related behaviours, well-being, and professional development are interrelated within nursing education. As a result, current evidence suggests that the relationship between PI and HL remains implicit rather than explicitly theorised.

The review further identified a tension between professional expectations and students’ reported health-related behaviours. While international standards and ethical frameworks emphasise HL and role modelling as professional responsibilities [13,14], empirical studies suggest that students often perceive HL as a personal choice rather than a professional obligation, with limited perceived impact on care quality [31,33]. This divergence appears to reflect a misalignment between formal professional values and students’ lived experiences during training. It is also reflected in accounts of moral discomfort and internal conflict, particularly among students who smoke, where professional role expectations seem to conflict with personal identity. Although such experiences are associated with feelings of stigma, they do not appear to consistently translate into behaviour change. This tension may indicate that expectations related to HL are not fully integrated into students’ understanding of professional responsibility. From an educational perspective, this raises questions about how HL is addressed within nursing curricula and whether sufficient support is provided for students to reconcile personal behaviours with professional expectations. In addition, the literature suggests that the ethical dimension of role modelling may remain implicit rather than explicitly discussed, potentially limiting students’ ability to critically engage with these expectations. Moreover, Aho et al. [31] draw attention to the role of the hidden curriculum in shaping students’ health-related behaviours, illustrating how nurses everyday clinical practices and the conduct of professional role models may implicitly normalise unhealthy coping strategies. This may further complicate the development of consistent professional values related to HL, as students are exposed to conflicting messages between formal education and clinical practice.

A healthy lifestyle is widely recognised as supporting long-term well-being, encompassing behaviours and habits that promote physical, mental, and social well-being [7]. However, this understanding appears to be in tension with nursing students’ reported health-related practices, as well as with their application of knowledge related to physiological functioning and self-regulation. The literature suggests that, despite awareness of the importance of HL, this knowledge is not consistently translated into everyday behaviour, particularly in the context of academic and clinical demands. Instead, unhealthy behaviours are described as being adopted as emotional coping mechanisms in response to stress [31,33], which may indicate that situational pressures outweigh knowledge-based intentions. This pattern underscores the importance of supporting students through the development of self-regulation skills and self-care, rather than relying on normative pressure or moralisation [32]. In a broader nursing context, insufficient self-care has been associated with reduced performance, an increased likelihood of errors, and potential risks to patient safety [18], suggesting that students’ health-related behaviours may also have implications for their future professional practice. This underscores its relevance not only as a personal practice but also as a professional consideration within nursing education.

In line with previously reviewed literature, more positive health-related orientations and better perceived health have been associated with a stronger professional self-concept, while a more developed PI appears to be linked to lower levels of stress, burnout, and maladaptive coping behaviours [39,41,42]. Building on this, the current review suggests that these associations may be better understood through underlying psychosocial processes rather than through direct behavioural regulation alone. Specifically, psychosocial aspects such as meaning in life, sense of coherence, and psychological capital are consistently identified as key mediating factors linking mental well-being and the development of PI [34,35,36,37,38]. These findings suggest that the relationship between HL and PI may be shaped primarily through psychological and existential pathways, including meaning-making, emotional regulation, and perceived coherence, rather than through explicit regulation of health behaviours. From this perspective, PI development in nursing education appears to be closely intertwined with the extent to which students are supported in their mental, existential, and psychological well-being. Constructs such as meaning in life, sense of coherence, and psychological capital have been described as fostering adaptive coping, emotional stability, and sustained engagement with the profession [34,35,37,38]. This may help explain why, despite awareness of HL, students may still engage in maladaptive coping behaviours under stress. This may further suggest that awareness of HL alone is insufficient, and that students may lack the knowledge, skills, or support needed to translate this awareness into effective self-regulation and behaviour that supports mental well-being.

Conversely, framing PI or HL narrowly in terms of individual discipline or behavioural compliance may risk overlooking these underlying processes. Such an approach may limit opportunities for students to develop effective self-regulation and meaning-making capacities, and may inadvertently contribute to the persistence of maladaptive coping strategies, such as smoking or problematic technology use [31,33,37]. Stress appears to function as a cross-cutting contextual condition within nursing education, shaping both PI development and health-related experiences. Previous research suggests that stress may have a dual role, with moderate levels supporting learning and professional growth, while excessive stress is associated with poorer mental well-being and challenges in identity development [40]. The present review extends this understanding by indicating that the effects of stress may be mediated through psychosocial resources such as meaning in life, sense of coherence, and psychological capital. When these resources are available, students may be better able to interpret and integrate challenging experiences constructively. In contrast, limited psychosocial resources may be associated with less effective coping, reduced well-being, and difficulties in engagement and professional development.

Taken together, the literature suggests that the relationship between PI and HL is characterised by complex and predominantly indirect interconnections rather than a simple bidirectional association. Rather than operating solely at the level of individual health behaviours, this relationship appears to be mediated through shared mental well-being-related and psychosocial processes and contextual influences. The tensions observed between knowledge, professional expectations, and students’ actual behaviours may be understood through students’ capacity for self-regulation, meaning-making, and coping within demanding educational and clinical environments. Processes such as emotional regulation, resilience, and supportive learning contexts may represent central mechanisms through which HL and PI develop in parallel and potentially reinforce one another.

### 4.5. Strengths and Limitations of the Review

This scoping review has several strengths that enhance the relevance and robustness of its findings. A key strength lies in its integrative examination of nursing students’ PI and HL as interrelated constructs. By synthesising empirical studies alongside review level evidence, the review offers a more comprehensive understanding of how HL is positioned within the development of PI, an aspect that has often remained implicit or fragmented in previous research. Methodologically, the systematic and transparent approach, including the use of multiple major databases spanning health and educational research, enhances the breadth and credibility of the synthesis.

Several limitations should be considered when interpreting these findings. First, although a comprehensive search strategy was applied across four major electronic databases, the number of studies meeting the inclusion criteria was limited, reflecting the current state of research at the intersection of PI and HL in nursing education. Due to the specific focus of this review, included studies were required to address both PI and HL among nursing students, even when the relationship between these concepts was indirect or not the primary focus. This may have reduced the number of eligible studies but was necessary to capture emerging and underexplored connections between these concepts.

Second, the search was restricted to these databases, and the inclusion of only open access, English language, peer-reviewed full-text articles may have limited the identification of potentially relevant studies, particularly those not freely accessible or published in other languages. This may have reduced the overall comprehensiveness of the review.

Third, the empirical evidence included in this review was geographically and methodologically concentrated. All quantitative studies were conducted in Asian contexts and involved 31 higher education institutions offering nursing education, whereas all qualitative studies were conducted in European settings. This regional and methodological clustering may influence the contextual scope of the findings and limit their transferability to other educational, cultural, and healthcare settings. This distribution may reflect differences in research traditions and educational priorities across regions, with qualitative approaches more prominent in European contexts and quantitative approaches more common in Asian settings. As this review is conducted from a European perspective, some culturally embedded understandings of HL and PI, particularly from non-European contexts, may not be fully captured. Quantitative studies in Asian contexts tended to involve larger samples, whereas qualitative studies in European settings were typically based on smaller samples, which may further reflect differing methodological traditions and a stronger emphasis on in-depth exploration of students’ experiences within European nursing education contexts.

Fourth, in line with scoping review methodology, no formal quality appraisal of the included studies was undertaken; therefore, the findings should be interpreted as a descriptive and exploratory synthesis rather than as an evaluative assessment of evidence quality. However, several methodological patterns were observed across the included studies and should be considered when interpreting the findings. These include variability in study design, relatively small sample sizes, and differences in how key concepts were conceptualised and operationalised. This heterogeneity may limit the comparability of findings across studies and contribute to variation in how the relationship between PI and HL is interpreted. In addition, relatively small sample sizes in some studies may reduce the robustness of the evidence and limit the transferability of findings across educational and cultural contexts. The frequent use of cross-sectional and self-reported data may further restrict insight into developmental processes and limit the interpretation of temporal relationships between HL and PI. Taken together, these methodological characteristics may contribute to the observed conceptual fragmentation and should be considered when interpreting the scope and implications of the findings.

Furthermore, the review focused exclusively on undergraduate nursing students, which constrains the extent to which the findings reflect the development of PI and HL at later stages of nursing education or professional practice, as well as in other health professions. An additional limitation concerns the heterogeneous conceptualisation of HL across the included studies. In many cases, HL was addressed indirectly through related constructs rather than being clearly and consistently defined. Although this conceptual heterogeneity enriched the analytical depth of the review, it limited direct comparability between studies. This may also highlight the need for further research within European nursing education contexts, where qualitative approaches are more prominent but large-scale and longitudinal evidence remains limited.

The identified relationships should therefore be interpreted with caution, as the available evidence is primarily exploratory. Given that the included studies were predominantly qualitative or cross-sectional and often addressed related but distinct constructs, the identified associations do not imply causal relationships but rather reflect patterns reported within the existing literature.

## 5. Conclusions

This scoping review provides a comprehensive synthesis of existing literature on the relationship between PI formation and HL among nursing students. Based on the included literature, the findings suggest that the relationship between PI and HL is complex, indirect, and not yet clearly conceptualised within nursing education research. Rather than being expressed through direct behavioural pathways, this relationship appears to be mediated primarily through mental well-being and related psychosocial aspects, as well as contextual influences within educational and clinical environments.

Both PI and HL are predominantly conceptualised as dynamic, relational, and contextually embedded processes that evolve throughout nursing education. However, HL is not consistently addressed as a distinct behavioural construct in relation to PI, but is more often examined through associated aspects such as self-care, coping, and well-being. At the same time, the physical dimension of HL remains comparatively underexplored within the context of professional identity development, with limited attention to concrete health-related behaviours such as physical activity, nutrition, sleep, and recovery.

These findings suggest a need for more integrative approaches in nursing education that explicitly address both behavioural and psychosocial dimensions of HL in relation to PI development. Future research should aim to develop clearer conceptual frameworks and adopt more methodologically balanced designs, including a stronger integration of qualitative and quantitative approaches. Although challenges related to nursing education and practice are global, the current distribution of studies suggests that in-depth exploration of students’ subjective experiences remains regionally concentrated. Expanding qualitative research across diverse cultural contexts, alongside larger-scale longitudinal and mixed-methods studies, may provide a more comprehensive understanding of how HL and PI are interrelated. Given the exploratory nature of the available evidence and the methodological limitations of the included studies, these findings should be interpreted with caution.

## Figures and Tables

**Table 1:** Overview of the characteristics of the included studies, listed alphabetically by first author.

Authors, Year	Study Design	Participants and Study Context	Keywords	Aims	Outcomes
Aho et al. (2019) [31]	Qualitative	Practical nursing students (n = 29) in Finland; vocational education; smokers	Smoking; identity; normalisation	Explore smoking and PI conflict	Smoking normalised; coping mechanism; identity conflict in PI
Damayanti et al. (2025) [32]	Quantitative (cross-sectional study)	Nursing students (n = 1071) in Indonesia; nursing education; high stress	Self-care; nursing students	Examine self-care practices	Self-care imbalance: higher emotional/spiritual, lower physical
Kelly et al. (2017) [33]	Qualitative (Delphi technique)	Nursing students, stakeholders (n = 25) in England, UK; policy and education context	Health behaviours; role model	Explore role model expectations	Role modelling expectations contested; limited support
Liu et al. (2025) [34]	Quantitative (cross-sectional study)	Nursing students (n = 286) in China; post-pandemic context	Sense of coherence (SoC), PI, academic emotion	Examine SoC and PI	SoC linked to PI via emotional and cognitive mediators
Qiu et al. (2023) [35]	Quantitative (cross-sectional study)	Nursing students (n = 418) in China; post-pandemic context	Meaning in life; PI	Examine sense of meaning (SoLM) and PI in the context of the pandemic	SoLM—perception of life as purposeful, coherent, and valuable; psychological resource supporting well-being and compassionate, holistic care; Meaning in life associated with stronger PI
Soerensen et al. (2024) [36]	Qualitative	Nursing students (n = 26) in Denmark; clinical and educational settings	PI; learning environment	Explore PI formation in context	Learning environment shapes PI; importance of belonging and safety
Ye et al. (2025) [37]	Quantitative (cross-sectional study)	Nursing students (n = 637) in China; high workload context in two medical universities	Smartphone addiction; PI; meaning in life (MiL)	Examine MiL and coping	MiL—perception of life as valuable and purposeful; internal experience of direction-seeking; MiL reduces maladaptive coping via PI
Zhang et al. (2025) [38]	Quantitative (cross-sectional study)	Nursing students (n = 1332) in China; transition to practice	Psychological capital; employment	Examine employment intention	Psychological capital supports engagement and transition

Note: MiL—meaning in life; PI—professional identity; SoC—sense of coherence; SoLM—sense of life meaning.

**Table 2:** Overview of the included review articles, listed alphabetically by first author.

Authors, Year	Review Type	Focus	Primary Studies	Outcomes
Alsararatee et al. (2025) [39]	Systematic Literature Review	Nursing student burnout, its effects on self-concept, engagement, and psychological health, and mitigation strategies	28 (4 RCTs, 15 non-randomised, 8 descriptive)	- Burnout associated with poorer professional self-concept, lower self-efficacy, reduced academic performance, and diminished psychological well-being; - Supportive learning environments and early resilience-based interventions as protective factors.
Araújo et al. (2023) [40]	Scoping Literature Review	Positive and negative aspects of stress experienced by nursing students during clinical practicum	32 (30 quantitative [27 cross-sectional, 1 comparative, 1 cohort, 1 exploratory], 2 qualitative)	- Both positive and negative effects of stress during clinical practicum; - Positive stress supporting learning and performance; - Negative stress impairing well-being, academic outcomes, and PI development.
Juanamasta et al. (2023) [41]	Systematic Literature Review	Factors related to the professional self-concept of nursing students and nurses	19 (cross-sectional)	- Professional self-concept influenced by organisational, individual, and emotional factors; - Organisational support, personal and health-related resources, and emotional competencies as key influences for both nursing students and practising nurses.
Xu et al. (2023) [42]	Literature Review	Professional self-concept: conceptualization, measurement tools, influencing factors, effects, and interventions	54 (predominantly quantitative; design not specified)	- Dynamic conceptualisation of professional self-concept influenced by individual and environmental factors; - Associated with positive academic, clinical, and mental health outcomes; supported by targeted educational interventions.

Note: RCT = randomised controlled trial.

**Table 3:** Conceptualisations of nursing students’ professional identity, healthy lifestyle, and their interrelationships.

Study	Conceptualisation of Professional Identity	Conceptualisation of Healthy Lifestyle	Interrelationships Between Professional Identity and Healthy Lifestyle
Qiu et al.(2023) [35]	Internalisation of professional role; self-belief and commitment	Psychological well-being via sense of life meaning (SoLM)	SoLM supports PI, coping, and well-being; low SoLM linked to poorer mental health and decision-making
Ye et al. (2025) [37]	Professional self-concept; commitment to development	Meaning in life (MiL); behavioural self-regulation; avoidance of addictive behaviours	PI mediates between MiL and coping; stronger PI reduces maladaptive behaviours
Damayanti et al. (2025) [32]	Self-development and “healing” role; PI linked to self-care	Holistic self-care (physical, mental, emotional, spiritual well-being, etc.)	HL supports PI through self-awareness and resilience; imbalance in physical self-care
Kelly et al. (2017) [33]	PI linked to professional values and role expectations	Behavioural HL (e.g., smoking, weight, activity)	Relationship contested; role modelling expectations vs. personal behaviour
Liu et al. (2025) [34]	Dynamic construct shaped by values and self-reflection	Salutogenic HL via sense of coherence (SoC); modifiable, health-promoting resource	SoC supports PI through emotional and cognitive pathways
Aho et al. 2019) [31]	Emerging, unstable identity; social and moral development	Smoking as coping and identity-related behaviour	Tension between PI and HL; identity conflict and hidden curriculum influence
Zhang et al. (2025) [38]	Internalisation of professional values and role	HL via psychological capital and health resources	Psychological capital supports PI, well-being, and career intentions
Soerensen et al. (2024) [36]	Relational, context-dependent identity formation	HL via ontological security and well-being	HL influences PI indirectly through safety, belonging, and emotional stability

Note: HL—healthy lifestyle; MiL—meaning in life; PI—professional identity; SoC—sense of coherence; SoLM—sense of life meaning.

**Table 4:** Healthy lifestyle aspects associated with professional identity in the included studies.

Healthy Lifestyle Area	Study	Healthy Lifestyle Aspects
**Mental well-being**-related aspects	Ye et al. (2025) [37]	Meaning in life; self-care; smartphone addiction
	Qiu et al. (2023) [35]	Meaning in life (SoLM)
	Liu et al. (2025) [34]	Sense of coherence
	Zhang et al. (2025) [38]	Psychological capital
	Soerensen et al. (2024) [36]	Ontological security; psychological well-being
**Physical and mental well-being**-related aspects	Damayanti et al. (2025) [32]	Holistic self-care (balance)
**Physical health behaviour**-related aspects	Kelly et al. (2017) [33]	Body weight; smoking
	Aho et al. (2019) [31]	Smoking (coping behaviour)

## Data Availability

All data supporting the findings of this study are available within the article.

## Supplementary Materials

The following supporting information can be downloaded at https://www.mdpi.com/article/10.3390/nursrep16040121/s1, Table S1: PRISMA_2020_checklist [30].

## Public Involvement Statement

No public involvement in any aspect of this research.

## Guidelines and Standards Statement

This manuscript was drafted against The PRISMA2020 statement: an updated guideline for reporting systematic reviews (See Table S1 in the Supplementary Materials).

## Abbreviations

The following abbreviations are used in this manuscript:

AACN	American Association of Colleges of Nursing
HL	Healthy lifestyle
ICN	International Council of Nurses
JBI	Joanna Briggs Institute guidance
MiL	Meaning in life
PI	Professional identity
RCT	Randomised controlled trial
SoC	Sense of coherence
SoLM	Sense of life meaning
WHO	World Health Organization

## Appendix A

### Appendix A.1. Scopus Search Strategy, December 2025

**Table:**

Search	Query	Records Retrieved
#1	(“nursing student *” OR “student nurse *” OR “undergraduate nursing student *” OR “baccalaureate nursing student *” OR “nursing education” OR “nurse educator *” OR “nursing educator *” OR “academic nurse *”)	286,048
#2	(“healthy lifestyle” OR “health behavior *” OR “health behaviour *” OR “health-promoting behavior*”) (“healthy lifestyle” OR “health behavior *” OR “health behaviour *” OR “health-promoting behaviour *” OR “health responsibility” OR “lifestyle behavior *” OR “stress management” OR “self-care”) OR (“healthy eating” OR “balanced diet” OR “nutritious diet” OR “nutrition behavior *”) OR (“physical activity” OR “exercise”) OR (“well-being” OR “wellbeing “ OR “wellness “) OR (“adequate sleep” OR “ rest “)	490,070
#3	(“professional identity” OR “nursing professional identity” OR “professional self-concept” OR “identity formation” OR “professional role identity” OR “role perception”)	127,028
#4	#1 AND #2 AND #3	371
	Limited: Document Types: Article or Review Article. Research Areas: Nursing. Languages: English. Open Access. Years 2015–2025	60

Note: The asterisk (*) denotes truncation, enabling retrieval of all word variants sharing the same root (e.g., “student”, “students”).

### Appendix A.2. MEDLINE (Via EBSCO) Search Strategy, December 2025

**Table:**

Search	Query	Records Retrieved
#1	(MH “Students, Nursing” OR MH “Education, Nursing” OR MH “Faculty, Nursing” OR TX “nursing student *” OR TX “student nurse *” OR TX “undergraduate nursing student *” OR TX “baccalaureate nursing student*” OR TX “nursing education” OR TX “nurse educator *” OR TX “nursing educator *” OR TX “academic nurse *”)	104,361
#2	(MH “Health Behavior+” OR TX “healthy lifestyle” OR TX “health behavior *” OR TX “health behaviour *” OR TX “health-promoting behaviour *” OR TX “health responsibility” OR TX “lifestyle behavior *” OR MH “Self Care+” OR TX “self-care” OR MH “Stress, Psychological+” OR TX “stress management” OR MH “Diet+” OR MH “Nutrition+” OR TX “healthy eating” OR TX “balanced diet” OR TX “nutritious diet” OR TX “nutrition behavior *” OR MH “Exercise+” OR TX “physical activity” OR TX “exercise” OR MH “Health Promotion+” OR TX “well-being” OR TX “wellbeing” OR TX “wellness” OR MH “Sleep+” OR TX “adequate sleep” OR TX “rest”)	2,288,476
#3	(MH “Professional Role” OR MH “Self Concept+” OR TX “professional identity” OR TX “nursing professional identity” OR TX “professional self-concept” OR TX “identity formation” OR TX “professional role identity” OR TX “role perception”)	156,474
#4	#1 AND #2 AND #3	997
	Limited: Academic Journals. Linked Full Text. Peer Reviewed. English Language. Past 10 years.	106

Note: The asterisk (*) denotes truncation, enabling retrieval of all word variants sharing the same root (e.g., “student”, “students”).

### Appendix A.3. Data Charting Form

**Table:**

Authors, Year of publication
Study design
Participants, study setting and context with country
Key concept
Objective
Outcomes
KEY FINDINGS
Conceptualization of professional identity
Conceptualization of healthy lifestyle
Interrelationships between professional identity and healthy lifestyle
Healthy lifestyle aspects associated with professional identity
